# Adulterations of Various Substances Used in Medicine and the Arts, with the Means of Detecting Them: Intended as a Manual for the Physician, the Apothecary and the Artisan

**Published:** 1847-02

**Authors:** 


### PART II.—REVIEWS.

### ARTICLE VIII.

*Adulterations of various Substances used in Medicine and the Arts, with the Means of Detecting them: intended as a Manual for the Physician, the Apothecary and the Artisan.* By Lewis C. Beck, M.D., Prof. of Chemistry in Rutgers College, New Jersey, and in the Albany Medical College: Honorary Member of the Medical Society of the State of New York, etc. New York: Samuel S. & William Wood, No. 261 Pearl Street. 1846. pp. 333. 12mo.

This manual is intended to exhibit the adulterations of substances used in the arts, as well as in medicine; and the processes for detecting them are generally quite simple, and may be easily followed by those who have but a slight acquaintance with chemistry. The style of the work is plain; technicalities are as little used as could be expected, in a work of this kind, on which account it is useful to a larger proportion of readers than it otherwise would be.

This simplicity of style would appear to be the more necessary, as it is intended for others than medical men.

In an appendix is a full description of the chemical operations, noticed in the body of the work, directions for the preparations of re-agents, and several tables showing the behavior of some of the re-agents with the more important substances. This addition adds materially to the value of the work.

We will select from it some portions which treat of the more important medicinal agents, such as are in every day use by the practitioner. We will commence with Columba, an article which is frequently adulterated with other roots differing greatly from it in medicinal properties.

"Colomba, U. S.—The root of the *Cocculus palmatus D. C.* (*Menispermum palmatum Lam.*), a native of Southern Africa. It is generally found in the shops in dried cylindrical slices, which have a thick yellow bark covered with an olive-colored skin, and a browner and spongy central portion. It is usually much worm eaten, and for use those pieces should be selected which are the least so. It ought to have a bright color. It is sometimes mixed with slices of bryony root, *Bryonia dioica*, a most dangerous adulteration, consisting in the substitution of a drastic purgative for a grateful tonic. The fraud probably originated in a belief which once prevailed that columba was the root of the *Bryonia epigæa W.* which is said to resemble it in its properties.—(*Burnetts Outlines of Botany.*)

"Columba, when moistened and touched with tincture of iodine, should become blackish in consequence of the presence of starch. False columba presents no change of colour when treated in this manner. Pure columba gives no colour to sulphuric ether; false columba gives it a fine yellow.

"The root of the American columba, *Frasera Walteri Mich.*, is said to have been sold in some parts of Europe for the genuine, and perhaps the same thing has been done in this country. According to M. Stolze of Halle, it appears that while the tincture of columba remains unaffected by the sulphate or sesquichloride of iron and gives a dirty grey precipitate with tincture of galls, the tincture of frasera acquires a dark green with the former reagent, and is not affected by the latter."

"Mercury, Protochloride of.—*Hydrargyri Chloridum Mite, U. S.—Calomel.*—This compound, when obtained by sublimation, occurs in a white, fibrous, crystalline mass, studded with shining, transparent crystals, having the shape of quadrangular prisms. When finely powdered, it acquires a buff colour. As formed by precipitation, it is a white, tasteless, inodorous and insoluble powder. Its specific gravity is 7.20. When heated to redness, it rises in vapour without previous fusion. By a moderate heat it becomes yellow, but recovers its white colour on cooling. When rubbed with the alkalies, or alkaline earths, it becomes black, in consequence of the formation of protoxide.

"Through carelessness in its preparation, calomel sometimes contains corrosive sublimate. This may be detected by heating a portion of the suspected calomel with eight or ten times its bulk of alcohol, and then adding to the alcoholic liquor some solution of ammonia, which causes a white precipitate if corrosive sublimate is present. The presence of chalk may be ascertained by the effervescence which follows the addition of a very small quantity of very dilute muriatic acid, and the precipitate produced in the clear neutral solution by oxalate of ammonia. To determine whether this is not caused by white lead, which may also be used as an adulterant, pass a stream of sulphuretted hydrogen through the filtered muriatic solution, when, if lead is present, a black precipitate will be produced. After the separation of the sulphuret by filtration, the clear solution may be tested for lime in the manner already pointed out. Nitrate of mercury may be detected by digesting the calomel in dilute nitric acid, and then adding potassa, when a red precipitate will be produced.

"Calomel changes colour by exposure to the light; it should, therefore, be kept in a dark place, or in bottles painted black, or covered with black paper."

In addition to the foregoing we would add iodide of potassium, which when added to water in which calomel containing corrosive sublimate has been digested, produces a scarlet precipitate. Care should be taken not to add it in excess, as it will cause the precipitate to disappear, by forming a double soluble salt, in which the iodide of potassium acts the part of a base, and the biniodide of mercury that of an acid. This is a very striking and delicate test. We might also add that of nitrate of silver, a pure solution of which, if added to water, digested upon calomel containing corrosive sublimate, will produce a white precipitate of chloride of silver. This also is capable of detecting a very small quantity of the bichloride of mercury.

It is very important that physicians should obtain a pure article of calomel, and the more so, both on account of its frequent use, and the highly deleterious properties that might be communicated to it, by one of those agents with which it is liable to be adulterated.

"Mercury, Protoxide or Black Oxide of.—*Hydrargyri Oxidum Nigrum, U. S.*—A dark grey or nearly black powder, insoluble in water and yet having a metallic taste. Its specific gravity is 10,69. When pure, it is entirely soluble in acetic and nitric acids, and entirely insoluble in muriatic acid which forms with it water and calomel. By a strong heat it is completely dissipated, and metallic mercury is sublimed.

"Protoxide of mercury has a great tendency to resolve itself into peroxide of mercury and metallic mercury, and it is extremely difficult to procure it in a state of absolute purity. It is often adulterated, especially when in the form of the blue mass or of ointment, with lampblack, sulphur and other substances; and sometimes this ointment is almost wholly free from mercury. Such frauds may be detected by melting the ointment, or by warming it in very strong alcohol, or in ether, which dissolve the fat, and deposit the mercury, if present, or demonstrate the deficiency when absent.

"The blue mass is sometimes prepared with conserve of roses, to which sulphuric acid has been added to heighten the colour; and it then contains subsulphate of mercury, which possesses very irritating properties. The presence of this salt may be detected by triturating the mass with boiling water and adding to the filtered liquor a solution of nitrate of baryta; if any sulphate be present, a white precipitate, insoluble in nitric acid, will be produced.

"If the protoxide of mercury is mixed with the peroxide, it may be detected by dissolving the suspected substance in muriatic acid and adding potassa to the filtered solution. A reddish or yellowish precipitate indicates the presence of peroxide. Oxalate of ammonia added to this solution will cause a white precipitate if the protoxide contains lime. Calomel, if present, may be detected by boiling the powder with a solution of potassa, which, when the solution is saturated with nitric acid, will give a precipitate of chloride of silver on the addition of the nitrate of that metal.

"The presence of metallic mercury may be detected by the protoxide not being completely soluble in acetic acid."

We will next notice the article of milk. The remarks in relation to it will be found more particularly useful to such as live in large towns and cities, and who are dependent upon others for their supply. The physician is also much interested in this matter, as, undoubtedly, in large towns many of the dieases of children are produced, and previously existing ones much agravated, by milk of a depraved quality.

"Milk.—In large cities, where the consumption of milk is very great, it is seldom exposed for sale without having received some fraudulent addition. Although these adulterations are not always positively injurious, they at least diminish those qualities which render milk so valuable an article of food. Many of the frauds praticed with it in Paris and other European cities, have probably never been here introduced: but there are some facts in regard to this liquid which deserve to be noticed in a work of this kind.

"Milk is a white opaque liquid, yielding about ten or twelve per cent. of solid matter on being evaporated to dryness by a steam heat. Its specific gravity is about 1,030. It is usually slightly acid, as may be seen by its reddening litmus paper; but occasionally it is an alkaline. When diluted with water it presents a bluish colour, instead of that dull white, which is characteristic of the pure substance.

"Various instruments have been invented for the purpose of determining in an easy and certain manner the comparative goodness of samples of milk. They are known by the names of *Lactometers*, *Creamometers*, *Galactometers*, and *Lactoscopes*. Some of these are similar in construction to the hydrometer, and their employment is founded upon the assumption that the specific gravity of milk is a true criterion of its richness and purity. But it is a serious objection to such instruments that the milk of different cows varies in its density, and that the food, the age and state of health of the animal also effect considerable changes in this respect. It is this variation in the specific gravity of pure milk which renders it difficult to detect even considerable additions of water. From the researches of Lassaigne it appears that mixtures of milk and water, in which the proportion of the latter is less than one-fourth or one-third, have a density not sufficiently reduced below the *minimum standard* of pure milk, to be determined by the lactometer.

"The *Creamometer* consists of a glass tube about a foot long and half an inch in diameter, supported upon a foot. If milk is poured into such a tube and permitted to repose there, the cream which it contains rises to the surface and forms a cake, the bulk of which, when compared to the bulk of the milk, denotes the comparative goodness of the sample. The tube should be graduated into ten parts, and the two upper parts divided each into ten others. It is then easy to determine at a glance the *per centage* of cream contained in any sample of milk submitted to trial. It should be previously ascertained how many parts of cream are contained in one hundred parts of genuine new milk. Some hours are required to perform an experiment with this instrument, and this seems to be the principal objection to its general employment.

"Among the adulterations of milk that have been noticed, are the addition of flour of various kinds, starch, white and yolk of eggs, gum arabic, dextrine, emulsions of various seeds, chalk, plaster of Paris, bicarbonate of soda, and even the cerebral matter of the sheep. Many of these may be easily detected, as flour and starch, by the blue colour which they give on the addition of the tincture of iodine. The presence of eggs may be ascertained by filtering the milk and then boiling the filtered liquor, when flocks of albumen more or less abundant will be formed. Gum arabic, unless added in large quantity, has very little effect upon the density of milk, but such addition is too expensive to be advantageously practiced. The taste of dextrine is too disagreeable to be employed as an adulterant. Chalk and plaster of Paris, if used, will subside upon setting aside the milk and can be then detected by the appropriate tests.

"There is no doubt that these supposed adulterations have been greatly exaggerated, as most of the substances are easy of detection, and do not after all answer the purpose for which they are said to be employed. The frauds which are known to be extensively practised with milk, are the removal of the cream, dilution with water, and the addition of bicarbonate of soda to neutralize its acidity. I have already referred to the difficulty which attends the detection of water in milk unless added in the proportion of a fourth or a third part. The peculiar color of such diluted milk would be a good test, if it were not that expert *manufacturers* add some coloring material, as annotto, the juice of the carrot, turmeric, &c., to conceal the change in this respect. The presence of bicarbonate of soda may be ascertained by its effervescence on the addition of an acid, as it is generally added in excess. Cases are referred to in which this salt has been used in such quantity as to cause the death of children who have used milk thus adulterated.—(*Garnier and Harel, Des Falsifi. des Subs. Alimentaires.*)

"But the deterioration which milk suffers from other causes is equally worthy of consideration. I refer to the effects produced upon this liquid by the different influences to which the animal is subjected, as food, state of health, fatigue, &c. It is well known that various articles eaten by cows pass rapidly into the circulation and can be detected in the milk. Thus, milk has a yellow colour when cows eat the marsh marigold (*Caltha palustris*); a bitter taste, when they eat wormwood; and the strong taste and colour of garlick, when they pasture in fields where this plant is abundant. The purity and healthfulness of milk must, therefore, depend very greatly upon the food with which cows are supplied. The diseases of these animals, whether induced by improper food or otherwise, must also have an important influence upon the quality of their milk. The remarkable milk sickness of the Western States affords a stiking illustration of the correctness of these statements. It is not without reason, therefore, that the great mortality among children in Paris is ascribed chiefly to the bad quality of the milk with which such a large number are constantly fed. It is said that the cows whose milk is intended for infants, are kept in close and warm stables, for the purpose of increasing the quantity of the liquid. But tubercles have been found in the lungs of almost all these cows in Paris and its vicinity.—(*Garnier and Harel.*)"

"Nitrous or Hyponitrous Ether.—*Sweet Spirit of Nitre,* (*Spiritus Ætheri Nitrici, U. S.*,) is a mixture of nitrous ether and alcohol in variable proportions. It is a colourless volatile liquid, with a fragrant ethereal odour and a pungent, aromatic, sweetish taste. It boils at about 160°, is very inflammable and burns with a whitish flame. Its specific gravity is 0.834 U. S., to 0.847 Ed. It generally reddens litmus slightly, but when perfectly pure should be without any acid reaction, and should volatilize without leaving the slightest residue.

"This article is very extensively adulterated. Wholesale dealers usually keep two, or even three, qualities of this preparation; the inferior ones being obtained by diluting the best with different quantities of water, or spirit of wine and water. 'Some years since large quantities of spirit of wine, flavored with hyponitrous ether, were imported into London, under the name of spirit of nitric ether, in order to avoid the duty payable on it as spirit of wine.'—(*Pereira.*) The extent of this mixture can sometimes be determined by its specific gravity, but at others it is a fallacious criterion.

"Sweet spirit of nitre often contains acetic acid or some of the acids of nitrogen in such qnantity, as to redden litmus strongly and to cause an effervescence when added to carbonate of potassa. If a deep olive colour is produced with the protosulphate of iron, it indicates the presence of a nitrogen acid. The presence of these acids increases the specific gravity of the sweet spirits of nitre; but the addition of hyponitrous ether produces the same effect. A specific gravity lower than the U. S. standard would probably show that the alcohol is stronger than it should be, and either in the proper amount or in too large proportion.

"When the specific gravity of sweet spirit of nitre is as high as that assigned by the Edinburgh College, the fraudulent addition of water and alcohol may be detected by agitating it with twice its volume of concentrated solution of chloride of calcium. If the article is of full strength, twelve per cent. of ether will slowly separate, which is twelve twentieths of the quantity present. If less ether separates, it shows the presence of too much water and alcohol. This test, however, is said not to be applicable to the U. S. preparation.—(*U. S. Dispensatory.*)"

"Opium.—*Opium, U. S.*—This is the name given to the inspissated juice which flows from incisions made in the unripe capsules of the *Papaver somniferum L.* It is only effectively obtained in warm climates, and the chief supply is procured from Turkey and India. It is a very heterogeneous compound, consisting of at least a dozen proximate principles, of which the most important are, *Morphine*, *Narcotine* and *Codeine*.

"Several varieties of this drug are described by writers on the Materia Medica. Our market, however, is almost exclusively supplied with the *Turkey Opium*, which is chiefly shipped from the port of Smyrna, and hence often called *Smyrna Opium.* It occurs in irregular masses, more or less flattened, covered with pieces of the dried leaves and with the reddish winged capsules of some species of *Rumex*, and sometimes also with poppy leaves. It has a rich reddish brown or fawn colour, a waxy lustre when cut, a tough consistence and a tolerably compact texture. Its odour is heavy, strong and narcotic; its taste bitter, arid and nauseous.

"There is no article in which frauds have been more extensively practiced than in opium. Even the Turkey opium, the best kind in market, one-fourth part generally consists of impurities. Among the substances employed as adulterants are, the extracts of the poppy, lettuce, and liquorice, gum Arabic, gum tragacanth, aloes, the seeds of different plants, sand, ashes, small stones and pieces of lead. An Armenian, who had been for many years engaged in the extraction of opium, informed Mr. Landerer, of Athens, that not a single cake of opium comes from the East without having been mixed in the soft and fresh state with grapes freed from their seeds and crushed. Another adulteration was said to consist of the epidermis of the capsules and stalks of the poppy, pounded in a mortar, and mixed with white of egg.—(*The Chemist*, *Sept.*, 1843.) Some samples of opium, also, which are apparently pure, are found to be totally destitute of the active principle of the drug.

"It is by no means easy, except by actual trial upon the system, to determine the quality of a sample of opium. The proportion of water may be judged of by the consistence, or, more accurately, by the loss on drying of a given weight of the drug. Many of the other impurities may be detected by a careful inspection. But the only certain test of the goodness of opium is to ascertain the proportion of morphine which it contains. This can only be accurately done by carefully following out some of the processes for the preparation of morphine. A pound of good opium should yield about a sixteenth of its weight of this alkali.—(See *Graham's Chemistry*.)"

"Peruvian Bark.—Under this general term are included the barks of various species of *Cinchona*, among which may be enumerated, as those of most frequent occurrence in market, the *Cinchona Calisaya*, or *Yellow bark*, *Cinchona rubra*, or *Red bark*, and the *Cinchona corona*, or *Pale bark*. Besides these there are several other kinds, but there is still much uncertainty in regard to their true botanical characters. It is well known, however, that their medicinal powers depend upon the alkaline principles which they contain. The most remarkable of these is *Quinine* or *Quinia*, the salts of which have almost entirely superceded the use of the powdered barks. Of this active principle the different varieties of Cinchona are known to yield very different proportions, and upon this circumstance their relative value depends.

"The frauds that are practiced with reference to Cinchona bark principally consist in the substitution of the inferior true barks for the finer kinds; the admixture of bark which has been exhausted by successive macerations and then dried, with good bark; and the substitution of spurious Cinchona barks for the true one. Of the false barks, three in particular have been described, viz: Piton bark, Caribean bark and Pitaya bark. They have all a disagreeable, bitter taste, not aromatic. The other adulterations, however, are very difficult of detection, as it is almost impossible to judge of the quality of bark (especially if in powder) by its physical properties. The quality of the yellow bark is best determined by the proportion of quinine which it yields. As the process employed is difficult of application on a small scale, the Edinburgh College has given the following test, by which the greater part of the alkali contained in a sample can be readily procured in an impure state: A filtered decoction of one hundred grains in two fluid ounces of distilled water gives, with one ounce of concentrated solution of carbonate of soda, a precipitate, which, when heated in the fluid, becomes a fused mass, weighing when cold two grains or more, and easily soluble in solution of oxalic acid. Manufacturers of disulphate of quinine generally, however, employ the test proposed by Guibourt, by which the quantity of lime contained in a sample is determined, it having been ascertained that those barks which are most rich in quinine also contain most lime. The process is as follows: 'Mix the bark in fine powder with water so as to form it into a fine paste; place this on paper, filter and add sulphate of soda to the filtered liquor as long as the white sulphate of lime is precipitated.'—(*Neligan.*)"

In addition to the above we would add that of tannic acid which has a powerful affinity for the vegetable alkalies, and when added to an infusion of bark, containing kinate of quinine in solution, it immediately unites with the alkali, forming an insoluble tennate, which forms a copious flocculent precipitate. If this is not produced the bark may be considered as destitute of quinine. This is a very delicate test, and can be easily applied by any person.

"Quinine or Quinia.—This is one of the vegeto-alkalies usually obtained from the yellow bark, *Cinchona Cordifolia Mut.* When pure, it occurs in white needle-form crystals, which are intensely bitter, soluble in 200 parts boiling water, almost insoluble in cold water, and highly soluble in alcohol and ether. It forms salts with acids, which are generally crystallizable. It is not used in its uncombined form. It is liable to be mixed with cinchonine, for which the tests will be found in the next article.

"Quinine, Disulphate of.—*Quiniæ Sulphas, U. S.—Sulphate or Subsulphate of Quinine.*—This salt, which is now largely manufactured, crystallizes in delicate white needles, which are very light and have the appearance of amianthus. It is efforescent, intensely bitter, requires about 740 parts of cold, and 30 parts of boiling, water for solution. It is soluble in 60 parts of cold alcohol, but in much less of hot. Very soluble in dilute sulphuric acid. It possesses the remarkable property of becoming luminous when heated a little above the boiling point of water, especially when rubbed.

"In consequence of the value of this salt it is often adulterated with various substances, as sugar, gum, starch, stearine, ammoniacal salts, sulphates of lime and magnesia, salicine and cinchonine.

"*Detection of Sugar, Gum and Starch.*—Sugar may be detected by dissolving the suspected salt in water, and adding precisely so much carbonate of potassa as will precipitate the quinine. The sugar may be separated by evaporating the filtered solution to dryness, and dissolving the residue by boiling alcohol. Gum and starch are left when the impure disulphate of quinine is digested in strong alcohol.

"*Detection of Ammoniacal and Earthy Salts.*—If ammoniacal salts are mixed with the disulphate of quinine, the fraud may be detected by the odour of ammonia which is given out when the mixture is put in a strong solution of potassa. And if the suspected disulphate is subjected to a red heat in a platinum spoon, the residue, if any, consists of earthy matters, the precise nature of which may, if necessary, be determined by subsequent trials.

"*Detection of Stearine.*—The presence of stearine may be ascertained by treating the suspected disulphate of quinine with water acidulated with muriatic acid. The disulphate is dissolved, but not the stearine. If the mixture is heated, the stearine swims on the surface of the liquor, and forms little transparent drops, which become opaque when the liquor cools.

"*Detection of Salicine.*—Recently, disulphate of quinine has been largely adulterated with salicine. The presence of this substance, when in considerable proportion, can be easily detected by the addition of sulphuric acid, which produces a fine red colour, while with the disulphate this acid forms a colourless solution. Where only a small proportion of salicine is mixed with the disulphate the following process may be employed: Pour on two parts of the disulphate twelve parts of concentrated sulphuric acid. The salt will be dissolved and coloured brown. Add 250 parts of distilled water; the brown colour disappears, and the salicine remains white and suspended in the liquid. The salicine is not dissolved by this acid solution of sulphate of quinine. Filter and collect on a glass, and the white bitter powder will give, with cold concentrated sulpuric acid, the vivid red reaction. The water should be added in small quantities at a time, and we should cease to add it when a precipitate is obtained which separates with facility.

"*Detection of Cinchonine.*—The samples of disulphate of quinine are now rarely free from the corresponding salt of cinchonine. Various processes have been suggested for the detection of the mixture. One of these depends upon the different action of chlorine upon the salts of quinine and cinchonine. If the disulphate of quinine be dissolved in a large quantity of chlorine water, and some water of ammonia is added, a deep green precipitate is formed, and the liquor also becomes intensely green. A salt of cinchonine similarly treated forms a dark red solution. Another mode consists in dissolving a portion of the mixed salt in water, and precipitating the solution with ammonia, collecting the precipitate, and boiling it in alcohol. If any cinchonine is present, it will be deposited in crystals as the liquor cools, while the sulphate of quinine remains in the mother liquor. According to M. Calvert, a saturated solution of disulphate of quinine in cold water gives, with a solution of chloride of lime, a precipitate, soluble in an excess of the latter; while a solution of sulphate of cinchonine of the same strength, treated in the same manner, gives a precipitate which is insoluble in a great excess of the reagent. The same effect is produced with lime water and solution of ammonia; and solution of chloride of calcium, while it furnishes a precipitate with a solution of sulphate of cinchonine, yields none with a solution of disulphate of quinine. For other distinctive characters of quinine and cinchonine, and their salts, see *Pereira's Materia Medica.*

"It should be added that the disulphate of quinine sometimes contains water in such an undue proportion as to constitute an adulteration. This is the case if one hundred parts of the salt lose more than eight or ten parts of their weight by the application of a gentle heat. Finally, to complete the catalogue of frauds, the bottles which come to us from France, purporting to contain an ounce of the disulphate, very rarely yield that amount; the reduction of weight being either the result of an understanding between the manufacturers and the wholesale dealers, or of the direct removal of a portion of the salt after it has been brought into this country."

"Silver, Nitrate of.—*Argenti Nitras, U. S.*—This salt, when pure, is in the form of white, tabular crystals, which ought to remain dry in the air, and when in a dry state ought not to blacken by exposure to the light. When heated on charcoal it deflagrates. It is soluble in its own weight of cold, and in half its weight of boiling, water. Upon adding muriatic acid to the solution, an abundant white precipitate is produced, which darkens by exposure to the light, is insoluble in nitric acid, but entirely soluble in ammonia.

"The *fused* nitrate of silver, or *Lunar Caustic*, is prepared by fusing the pure salt, and pouring it into heated moulds. In this state it should have the appearance of a grey or nearly white crystalline mass. It is usually, however, of a blackish colour, owing either to carelessness in the manufacture, or to the presence of organic matter, which under the influence of light causes a reduction of a portion of silver.

"Nitrate of silver is often largely adulterated. Thus it contains free silver from having been exposed to too high a heat, the nitrates of lead and copper, from the impurity of the silver used in its preparation, and chloride of silver from that of the acid. From fraudulent admixture, also, it is sometimes contaminated with nitrate of potassa.

"*Detection of Free Silver and Chloride of Silver.*—Dissolve the suspected lunar caustic in about its own weight of water. If it leaves more than a very slight dark residue, it contains either free silver or the chloride of silver. Add ammonia to this residue; the chloride of silver will be dissolved, while the free silver remains. From the ammoniacal solution the chloride may again be obtained by evaporation, and its amount determined. The free silver which remains after the separation of the chloride, may be dissolved in nitric acid and converted into a chloride by the addition of muriatic acid. Should any oxide of copper be contained in the insoluble residue, its presence will be detected by the blue colour caused on the addition of ammonia.

"*Detection of Nitrates of Copper and Lead.*—Dissolve a known quantity of the suspected nitrate in a large quantity of water, and after having separated the insoluble matter by filtration, add muriatic acid. All the silver will be thrown down as an insoluble chloride, while the chlorides of copper and lead, if the quantity of water is sufficiently large, will be held in solution. Filter the liquid, and then add sulphuric acid; if any lead is present an insoluble sulphate of lead will be formed. From the filtered solution copper may be precipitated, if present, as deutoxide, by caustic potassa. The precautions which are required to effect this separation are described in the article Silver.

"*Detection of Nitrate of Potassa.*—To detect this salt, lunar caustic is to be dissolved in water, and the solution precipitated by muriatic acid as in the process for the detection of the metallic nitrates. The filtered solution is then to be neutralized with ammonia and treated with sulphuretted hydrogen to separate any metals that may be present. The filtered solution, if it contains nitre, will, on evaporation, leave a salt easily recognized by its properties as a nitrate. If thrown upon burning coals it will deflagrate and leave a fixed residue.

"By operating upon a given weight of lunar caustic the proportions of the above adulterants may be easily determined. The real value of any sample may be accurately ascertained by dissolving it in water, precipitating it by muriatic acid, and determining the proportion of pure nitrate of silver by the weight of the dried and slightly heated chloride of silver. 100 parts of the nitrate of silver should give nearly 85 of the chloride of silver. An American dollar will yield nearly one ounce and a quarter of pure nitrate of silver."

We will next notice what he says in relation to wine, an article that is largely adulterated. When pure it may frequently be used as a medicinal agent, with much benefit; but as generally found in market, it cannot be depended upon, for which reason, it is not ordered as much as it otherwise would be.

"WINE.—*Vinum, U. S.*—Wine is a transparent liquid, of a yellowish, reddish yellow, or deep red colour. It consists principally of water and alcohol; but a great number of other substances are also found in it, as sugar, fecula, gluten, extractive, colouring matter, tannic acid, bitartrate of potassa, tartrate of lime, volatile oil, œnanthic ether, &c. It is upon the volatile oil that the peculiar taste and odour of wine, called the *bouquet*, is supposed to depend. The proportions of the ingredients vary greatly in the different kinds of wine. Tannic acid and colouring matter are in larger quantity in the red wines than in the white, while the alcohol ranges from nine or ten to twenty-five per cent.

"Numerous frauds are practiced with wine, and it is to be regretted that chemistry does not yet furnish the means of detecting them with all certainty. The most common of these are the mixture of wines of different vintages, and the addition of water, of brandy, of colouring matters, and of various saline substances.

"*Colouring Matters.*—The colour of red wines is due to the skins of the red raisins, with which the *must* is fermented. These wines also derive from this source the tannic acid to which they owe their astringent taste and their change from a red to a brownish black colour, upon the addition of a soluble persalt of iron.

"Among the substances said to be used for the purpose of giving a red colour to wines, are logwood, Brazil-wood, beet root, and the fruit of the elder and of the sloe. The process most to be relied on for determining their presence is that of Nees d'Esenbeck. It consists in dissolving one part of alum in eleven parts of distilled water, and one part of carbonate of potassa in eight parts of water. The suspected wine is mixed with an equal volume of the solution of alum, which renders its colour more brilliant; to this mixture the alkaline solution is then added, little by little, taking care not to decompose all the alum. The alumina is precipitated with the colouring matter of the wine in the form of a lake, which, both before and after the addition of the potassa, assumes tints which vary with the nature of the colouring matter. In pure wine the solution of alum produces a lake of a dirty grey colour, which an excess of the alkali partly dissolves, leaving the residue of an ash grey colour. If the wine has been coloured by any of the substances above mentioned, the precipitate left, after following out this process, is some shade of blue, violet, or rose.

"*Detection of Carbonates.*—The carbonates of lime, potassa and soda are sometimes employed to neutralize the acidity of certain wines. The first of these may be detected by evaporating a portion of the wine to about one-eighth its volume and adding to the residue twice its volume of alcohol of sp. gr. 0.921. By this means the sulphate and tartrate of lime, which naturally exist in the wine, are precipitated, and the acetate of lime dissolved. The solution is then filtered, and carefully evaporated to dryness. The filtered solution of this residue in water gives an abundant white precipitate with oxalate of ammonia, and evolves the odour of vinegar when decomposed by sulphuric acid.

"Carbonate of potassa is sometimes added to wine for the purpose of stopping the fermentation and of saturating the acetic acid which it may contain in excess. In this case acetate of potassa will exist in the wine. To determine the presence of this salt, evaporate a portion of the wine to a syrupy consistence and agitate the residue for some minutes with a small quantity of alcohol of the sp. gr. of about 0.842; upon the application of heat the acetate of potassa will be dissolved. The liquid after filtration is divided into two parts: the one is treated with a solution of the chloride of platinum, which gives with potassa a yellowish precipitate; the other is evaporated to dryness and the residue moistened with strong sulphuric acid, which liberates acetic acid, known by its peculiar odour. It should be stated that pure wine always contains acetate of potassa, but the quantity is so minute that these tests produce it with scarcely perceptible results.

"When wine is saturated with carbonate of soda, the tests employed for acetate of potassa give only negative results. In this case, the residue of the evaporation should be treated with alcohol of the sp. gr. of 0.920, which dissolves the acetate of soda. This solution is then evaporated, the residue dissolved in water, the solution filtered and slowly evaporated. If acetate of soda is present, crystals will be formed which have a sharp and slightly bitter taste, and are decomposed by strong sulphuric acid.

"*Detection of Lead.*—The addition of white lead or litharge to sour wines, to neutralize their acidity and to render them sweet, was formerly often practised, but is now rarely resorted to. The presence of lead in wine may be detected as follows: Evaporate a portion of the suspected liquor to dryness in a Berlin ware capsule, collect the dry residue and heat it to redness. Triturate the coaly mass with twice its weight of nitrate of potassa, and throw the mixture, a little at a time, into a small porcelain crucible heated to redness. The nitrate of potassa causes the combustion of the charcoal, and a fused mass, which contains the lead, remains. If the matter retains a deep brown colour, the ignition may be repeated with another portion of the nitrate. The residue is then treated with water acidulated with nitric acid, until it is completely dissolved. This solution is then filtered, and if it contains a salt of lead it will give a white precipitate on the addition of a few drops of sulphuric acid; a yellow precipitate with solution of chromate of potassa; and a black precipitate with sulphuretted hydrogen or hydrosulphuret of ammonia.

"*Detection of Alum.*—Alum is sometimes added to wine for the purpose of heightening its colour and of giving it an astringent taste. If the wine is red, mix it with a sufficient quantity of animal charcoal previously well washed with muriatic acid. When it has been deprived of its colour, filter, evaporate the filtered liquor to one-third of its volume, and then filter it a second time to remove the coloured precipitate which has been formed during the evaporation. If alum is present, the solution will have an astringent taste, and will give, upon the addition of potassa, a white precipitate soluble in an excess of the alkali; it will also cause, with chloride of barium, a dense white precipitate, insoluble in nitric acid.

"*Detection of Brandy.*—The addition of brandy to poor wines is often practised for the purpose of giving them the more strength to resist decomposition. If the proportion of brandy is large, and the mixture recent, its presence can sometimes be detected by its deflagration when thrown into the fire; but when the mixture is of long standing, this test is of no use. The proportion of alcohol in wine can be determined by distillation, but we have no means of distinguishing that which belongs naturally to the wine from that which has been added as an adulterant."

J. McL.

